# Comparing the effect of 0.06% -, 0.12% and 0.2% Chlorhexidine on plaque, bleeding and side effects in an experimental gingivitis model: a parallel group, double masked randomized clinical trial

**DOI:** 10.1186/s12903-017-0400-7

**Published:** 2017-08-18

**Authors:** Maliha Haydari, Ayse Gul Bardakci, Odd Carsten Koldsland, Anne Merete Aass, Leiv Sandvik, Hans R. Preus

**Affiliations:** Department of Periodontology, Institute of Clinical Odontology, Faculty of Dentistry, University of Oslo, Oslo, Norway

**Keywords:** Anti-plaque agent, Dental plaque, Gingivitis, Chlorhexidine

## Abstract

**Background:**

Chlorhexidine is the gold standard of dental plaque prevention. The aim of the present study was to compare the plaque and gingivitis inhibiting effect of commercial products containing 0.2%, 0.12% and 0.06% chlorhexidine in a modified experimental gingivitis model.

**Methods:**

In three groups of healthy volunteers, experimental gingivitis was induced and monitored over 21 days and simultaneously treated with the commercial solutions containing 0.2%, 0.12% and 0.06% chlorhexidine. The maxillary right quadrant of each individual received mouthwash only, whereas the maxillary left quadrant was subject to both rinsing and mechanical oral hygiene. Compliance and side effects were monitored at days 7, 14, and 21. Plaque and gingivitis scores were obtained at baseline and day 21.

**Results:**

The commercial mouthwash containing 0.2% chlorhexidine resulted in statistically significantly lower plaque scores than the 0.12 and 0.06% mouthwashes after 21 days use, whereas no statistically significant difference was found between the effects of the two latter.

**Conclusion:**

A commercially available mouthwash containing 0.2% chlorhexidine had statistically significant better effect in preventing dental plaque than the 0.12% and 0.06% solutions.

**Trial registration:**

ClinicalTrials.gov NCT02911766. Registration date: September 9th 2016.

**Electronic supplementary material:**

The online version of this article (doi:10.1186/s12903-017-0400-7) contains supplementary material, which is available to authorized users.

## Background

Chlorhexidine (CHX) is a bis-biguanide with documented bacteriostatic and bactericidal effects, on both Gram positive and - negative bacteria [1], fungi and some lipophilic viruses [2]. In the 1970’s CHX was studied and recommended by researchers as part of the prevention and therapy of periodontal diseases [3] because of its plaque inhibitory effect [4–7]. Besides its proven immediate bactericidal effect, chlorhexidine binds to the oral mucosa from which it is slowly released, prolonging its antibacterial effect [4, 8].

In Norway, CHX has mainly been marketed as a 0.2% non-alcohol solution, but recently a 0.12% mouthwash has also been approved. These two CHX mouthrinse formulations are only recommended for short term use, i.e. for patients that – for one reason or the other - cannot keep their mechanical tooth cleaning up to standard. A 0.06% solution, for daily use, has also recently been approved for the Norwegian market, claiming in ads (no references displayed) prevention of gingival problems and that it reduces the amount of plaque 3.5 times compared with mechanical tooth cleaning.

Only few studies have compared the effects of 0.2% and 0.12% CHX on periodontal indices. A systematic review [9] included 10 publications, and concluded that 0.2% CHX had a slightly better effect than 0.12%, but the practical, clinical implication of this finding was regarded as uncertain. Nearly all of the included articles in this systematic review [9] had applied the plaque index of Quigley and Hine [10] – or the Turesky modification [11] of this index. Since these indices include disclosing solutions and also variably register the protein coating of teeth, one should also test the efficacy of the CHX concentrations using other scoring indices, like the Løe & Silness’ [12] which only scores dental plaque. Moreover, to help the clinicians in their selection of the most effective plaque-preventing mouthwash when new products are presented, the actual commercial products should be tested, because added ingredients for commercially motivated enhanced taste, flavor and color may reduce the effect of the highly reactive CHX molecule. Based on a working hypothesis of 0.12% and 0.2% CHX having equal plaque-preventing effects and 0.06% CHX having a comparatively less efficacy, the aim of the present study was to compare the efficacy of the 2, in Norway, newly marketed (0.12% and 0.06% CHX), and the already well known mouthwash (0.2% CHX) – on plaque and gingivitis using both the Turesky modification of the Quigley and Hine plaque index [11] and the plaque and gingival index of Løe and Silness [12], as well as reporting on the short-term side effects.

## Methods

The present study was designed as a parallel group, double masked, randomized clinical trial. The experimental gingivitis model [13], with the modifications by Preus et al. [14, 15] was used to induce gingival inflammation under supervised conditions throughout the study. The Regional Committee for Medical Research Ethics, South East Norway, approved the study (REK 2016/1748). The U.S. National Institute of Health Clinical Trials Registry number is NCT02911766 (http://www.clinicaltrials.gov). The study adheres to the CONSORT guidelines.

**Fig. 1:**
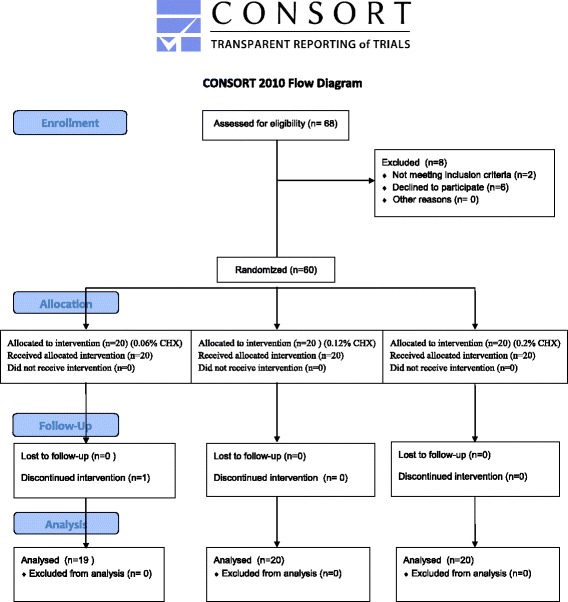
Patient flow diagram

The study population comprised 60 dental, medical, and dental hygienist students who volunteered to participate in the project. A meeting was arranged for the volunteers prior to the start of the study, through which the participants received information about oral rinsing products in general and CHX containing products as well as information on the study ahead, in particular. At this meeting 68 students showed their interest, but 6 withdrew because they realized that they had to abstain from tooth cleaning in quadrant 1 for 21 days. Two students were not eligible due to regular use of smokeless tobacco (Fig. 1).

Mean age of the participants was 21 years and 72% were females. The study period was 21 days in November 2016. All information, administration and data collection was performed at the Department of Periodontology, Institute of Clinical Odontology, Faculty of Dentistry, University of Oslo, Norway.

Inclusion criteria comprised healthy subjects of both genders, aged 18 years and older, having at least three of the following teeth in maxillary right and left quadrant: the canine, 1st bicuspid, 2nd bicuspid, 1st molar, healthy gingiva and periodontium. Exclusion criteria comprised smoking and/or use of non-smoking tobacco, pregnancy, lactation, any chronic diseases, clinical signs or symptoms of acute infection in the oral cavity, any prescribed or non-prescription systemic or topical medication except oral contraceptives, use of systemic antibiotics the last 3 months prior to the start of the study, history of alcohol or drug abuse or participation in other clinical studies in the last 4 weeks. Before inclusion, every participant signed an informed consent form in which anonymity was granted and confirmed.

The test solutions were the commercially available mouthwashes: 0.2% CHX,^1^ 0.12% CHX with 910 ppm NaF^2^ and 0.06% CHX with 250 ppm NaF.^3^ The three commercially available CHX mouthwashes were filled in identical, but differently labeled (A,B,C) bottles for blinding purposes. The 0.2% and the 0.06% CHX products were bought at a local pharmacy, whereas the 0.12% was donated to the project by the manufacturer.

Simple, restricted randomization was carried out using a computer generated random allocation Table [16] assigning the participants to the three study groups with 20 test subjects in each. They were all carefully instructed to rinse for 60 s. twice a day as recommended by the manufacturers.

Setting the baseline dental plaque score to zero was done by giving all participants a professional tooth cleaning with rubber cup, pumice paste and dental floss at the start of the study. The participants were given their test solution and subsequently instructed to rinse as described above. All information was given verbally as well as in writing.

Individual plastic tooth guards had been produced to fit the teeth in the upper right quadrant (Q1) [14, 15]. Together with this individual tooth guard, the participants were given identical prophylaxis packs containing a medium texture tooth brush, inter-dental floss and dentifrice and were instructed to insert the tooth guard in Q1 every time they brushed their teeth and to perform a mechanical oral hygiene routine twice daily in the three other quadrants. They should then rinse 30 s with tap water before and after removing the tooth guard. Following this procedure, the participants rinsed, as instructed, with the solution they randomly had been assigned, repeating the procedure for 21 days. Following the scoring at day 21, the participants received professional tooth cleaning after ending the study.

A team of five people were trained in the procedure of informing participants, receiving the test persons for evaluation, questionnaire and clinically monitoring them [15]. The principal investigator (HRP) and project managers (MH/AGB) managed all contact with the participants outside the scoring room. In between appointments the project team kept in touch with the test persons by text messaging and e-mail. The success of this service was evident by zero no-shows at the clinic.

At the interviews at day 7, 14 and 21 the project managers (MH/AGB) received reports from each participant about adherence to protocol as well as verbal complaints and descriptions of subjective side-effects. A special, assisted questionnaire had been prepared for these interviews. Reports of ill- and side effects were registered and categorized for later statistical evaluation. To investigate a possible recognition effect among the participants they were also asked if they had recognized the taste and knew (no guessing) which rinsing compound they were assigned to (Additional file 1, assisted questionnaire).

At day 21, the above mentioned interview was followed by an examination of clinical results. Before entering the scoring room MH/AGB advised the participants to refrain from any conversation with the scoring scientists inside, who had been instructed likewise. In the scoring room, two researchers (AMA, OCK) obtained the clinical data. Plaque index (PI) and gingival index (GI) [12] were recorded on the mesial, buccal, distal and palatal aspects of teeth 16, 15, 14, 13 and 23, 24, 25, 26. Adverse events like discoloration observed during the clinical examination (yes/no) and clinically visible oral mucosal reactions were registered. In addition the plaque index by Quigley and Hine, the Turesky modification [11] was finally registered. All clinical registrations were performed by the same experienced periodontist (AMA), leaving her colleague (OCK) to register recordings on specially designed charts. The clinical crew was kept blind to the group allocation of the participants at all times, as the statistician was the only one that had access to the code-book, and he did not participate in any clinical event.

### Statistics

The present experiment aimed at comparing the plaque and gingivitis preventing effect of the 0.12% CHX and 0.06% CHX solutions with the gold standard 0.2% CHX solution^4^ (no alcohol).

The total number of participants was 60, with 20 participants in each group. The number of participants was based on the following power calculation. The power analysis was based on the variable ‘average plaque score in each participant’ (APS). When comparing APS in two groups, a two-sided independent samples t-test was used, with 5% significance level. Average standard deviation in the 3 groups was 0.40. It may be shown that in order to have 80% test power to detect a mean difference in APS of at least 0.40 between two groups, at least 15 participants must be included in each group. Because some drop-outs were expected, it was decided to include 60 subjects in the study. Because 80% test power is generally accepted as sufficiently high in clinical studies, and the mean difference in mesial plaque score between group 1 and group 3 was 0.41, the above calculation suggest that our study had acceptable test power.

When comparing mean plaque score in two groups, a two-sided independent sample t-test was used, with a 5% significance level. When comparing proportion of subjects with a particular adverse effect, the “linear by linear association chi-square” test was used. The statistical analysis was conducted using the software of SPSS for Windows, Version 16.0 (SPSS Inc., Chicago, IL).

The distributions of the outcome variables were checked, and found to be sufficiently close to the normal distribution to allow for the use of a t-test.

## Results

From weekly reports and questionnaires, it was shown that 59 participants had followed the instructions diligently during the 21 days that the experiment lasted. One participant had violated the protocol and was excluded following the interview at day 14, resulting in a total study population of 59 persons at the final scoring, still leaving the sample size large enough for conclusions.

### Q1: Rinsing only quadrant

#### Plaque index [13]

Rinsing with 0.2% CHX resulted in an average plaque score of ***all surfaces combined, approximal surfaces only*** or ***mesial, buccal, distal surfaces together*** (i.e. the palatal surfaces taken out) after 21 days, which was statistically significantly lower (*p* < 0.05) than the results in the two other groups, the latter with no statistically significant difference between them (Table 1).

**Table 1:** Plaque Index (Loe & Silness) after three weeks – rinsing only quadrant (Q1) as well as rinsing + brushing quadrant (Q2)

	Rinsing only quadrant (Q1)					Quadrant 2 (Q2)
	Buccal/palatal	Proximal	Palatal	b+m+d	All surfaces combined	All surfaces combined
0.06% CHX	0.61 ± 0.43	1.24 ± 0.45	0.45±0.27	1.09 ± 0.50	0.93 ± 0.41	0.17± 0.30
0.12% CHX	0.63 ± 0.51	1.36 ± 0.56	0.37±0.38	1.20 ± 0.61	0,99 ± 0.53	0.24± 0.48
0.20% CHX	0.40 ± 0.39	0.90 ± 0.52*	0.32±0.44	0.75 ± 0.49*	0.65 ± 0.42*	0.14± 0.31

*statistically significant p<0.05

*b* buccal, *m* mesial, *d* distal

Twenty-one days of rinsing with these three commercial CHX products produced no statistically significant difference between the groups regarding the plaque scores on the ***buccal and palatal surfaces combined*** or the ***palatal surfaces alone*** (Table 1).

#### Gingival index [12]

When mouth rinse was the only plaque-inhibiting procedure used, the gingival index scores produced no statistically significant differences among the three groups after 21 days, neither as an average of all sites or approximal, buccal and palatal sites separately (Table 2).

**Table 2:** Gingival index (Løe & Silness) and plaque score (Quigley & Hine) in Quadrant 1 (rinsing only quadrant) and Quadrant 2 (Brushing & rinsing quadrant)

	Number of participants	Gingival index all sites combined Quadrant 1	Gingival index all sites combined Quadrant 2	Plaque score Quigley & Hine Quadrant 1	Plaque score Quigley & Hine Quadrant 2
CHX 0.06%	19	1.1 ± 0.87	0.8 ± 0.78	2.6 ± 1.16	0.7 ± 0.78
CHX 0.12%	20	1.2 ± 0.87	0.7 ± 0.82	2.2 ± 1.05	0.8 ± 0.78
CHX 0.2%	20	1.1 ± 0.82	0.7 ± 0.88	2.1 ± 1.48	0.7 ± 0.75

No significant associations

#### Turesky Modified Quigley & Hine Index [11]

Results showed no statistically significant differences between the three solutions (Table 2).

### Q2: Rinsing and mechanical oral hygiene quadrant

In the quadrant where both mechanical and chemical plaque control were performed, no statistically significant difference was found between the three groups, neither by the Quigley & Hine [11] (Table 2) nor the Løe & Silness [12] (Table 1) plaque indices. A close to 0 plaque- and gingival scores were registered in all patients.

### Adverse effects

Subjective complaints of discomfort and registration of clinical adverse effects were registered at day 7, 14 and 21 in all three groups. There were no statistically significant differences in self-reported taste sensations, soreness of oral mucosa/tongue/gingiva, feeling of dryness or discoloration in the participants among the three groups (Table 3).

**Table 3:** Subjective side effects as reported by the participants

Mouthwash	Number of participants	Side effects as reported by participants. n (%)							
		B1	B2	B3	B4	B5	B6	B7	B8
0.06 CHX	19	1 (5.3)	10 (52.6)	10 (52.6)	4 (21.1)	5 (26.3)	7 (36.8)	4 (21.1)	10 (52.6)
0.12 CHX	20	5 (25)	11 (55)	7 (35)	11 (55)*	8 (40)*	7 (35)	5 (25)	14 (70)
0.2 CHX	20	4 (20)	15 (75)	4 (20)	13 (65)*	12 (60)*	9 (45)	2 (10)	15 (75)

*p<0.05

B1: Taste; Bad – nauseating

B2: Taste: too strong/bitter

B3: Taste: Good (“I like it”)

B4: Taste perturbation (loss of taste)

B5: Numb feeing in tongue and mouth

B6: soreness in tongue and mouth

B7: desiccating/dry feeling

B8: Subjective Discoloration

However, statistically significant differences were observed with “loss of taste” and “numb feeling”, where respectively 65% - 60%, 55% - 40% and 21% - 26% complained about “loss of taste” - “numb feeling” in respectively the 0.2%, 0.12% and 0.06% CHX groups (Table 3).

No clinical adverse effects like erosions of the oral mucosa were registered, except that slight discoloration of teeth was registered by the clinical research staff. However, no difference was recorded between the groups (Data not shown).

Raw data with explanations are displayed in Additional file 2.

## Discussion

The findings of the present study showed that CHX 0.2% proved statistically significantly better than 0.12% and 0.06% CHX in preventing supragingival plaque on the participants’ teeth after 21 days when applying the plaque score of Løe and Silness [12]. Moreover, between them, the 0.06% and 0.12% CHX products showed no statistical significant difference in plaque inhibition with any of the plaque indices [11, 12]. No statistically significant differences in gingival index between the groups were found after 3 weeks rinsing with the 3 different CHX solutions.

The finding of no significant difference in gingival index scores between the three mouthwashes, despite significant difference in plaque scores between the 0.2% and the 0.12%/0.06% concentrations warrants a discussion. No human is free of slight gingival inflammation, and most adults have a GI of approximately 1 following regular tooth cleaning. The average GI for quadrant 1 (rinsing only) after 3 weeks in group 1, 2 and 3 respectively were all 1.1 (all sites combined), and 1.2 (only approximal surfaces). These were in concert with findings following the “the non-cleansing period”, i.e. no chemical or mechanical plaque preventing procedures from the original experiment by Löe et al. [13] where the GI of maxillary premolars and molars were 1.01 and 1.1 respectively. Testing CHX 0.2% in a group of soldiers, Fløtra et al. 1972 [17] showed that average GI score was a little shy of 1 in the experimental group after 8 weeks whereas the plaque index was 0.8, the latter being in the range of observations from the present study. Fløtra et al. [17] used a laboratory produced CHX solution, whereas the present study tested the commercial products, and this may have had an impact on the effect of the CHX on plaque as well as the gingivitis (for discussion - see below).

CHX 0.12% and 0.2% mouthwashes are both recommended for short-term use, and for medicinal purposes, classifying the products as medical products. CHX 0.06%, recommended for long-term use, is intended to be a supplement to daily tooth cleaning/brushing and is therefore classified amongst the cosmetic and toiletry product lines with a market strategy aimed at preventing dental plaque at a population level. Gingivitis is the result of prolonged tissue-exposure to supragingival plaque [18], and even if the 0.06% CHX did not produce significantly more gingivitis than the 0.12% and the 0.2% CHX after 3 weeks, a remaining long standing plaque index of 1 will induce gingivitis in the long run [13, 17, 18]. This brings to the discussion a much more serious problem. A low-concentrate biocide like CHX is bringing a potential danger for resistance development in oral bacteria – but even more so – in the sewage microbiome. The increased use of CHX in oral and general health care [19–28] is today a clear and present danger for resistance development against this versatile antiseptic. The overall risk for acquired resistance to biocidal agents, such as CHX, is still considered to be small, provided the antiseptics are being used with care and in correct situations [29, 30]. However, several outbreaks have been reported associated with contaminated CHX solutions [31] which demonstrate the ability of bacteria to adapt to CHX [32]. Therefore, future research carries with it the obligation to explore the bacterial resistance development against CHX, and not only in the oral cavity. The sewage microbiome, influenced by the increasing influx of low concentrate CHX, will eventually and inevitably produce more CHX resistant bacteria that will transcolonize humans and their microbiomes.

Few studies have compared different CHX products [33–39]. However, a systematic review and meta-analysis [9] comparing studies on 0.2% and 0.12% CHX concluded: a “small but significant difference in favor of 0.2% CHX, but that the clinical relevance of this difference was probably negligible”. These studies, all but one, used Quigley and Hine plaque index (11), or the Turesky modification [11] to score the presence of plaque after the prescribed use of the tested CHX compounds. The Quigley and Hine, Turesky [11] modification index requires plaque disclosing solution, which precludes the registration between baseline and endpoint in case the participants should not brush their teeth in the meantime. Therefore, the present study was only registering the plaque and gingivitis indices at baseline and at termination of the study. Both this index (12) and the plaque and gingivitis index of Løe and Silness [12] were used for comparison to previous studies. A possible explanation for the results conveyed here being different from those previously published [9, 33–39] may be that this plaque index [11] also register some of the protein coating that retains the plaque disclosing solution and that it is difficult to distinguish and set a correct score, whereas the Løe and Silness plaque index [12] detects the plaque accumulation along the gingival margin. This notion was supported by the high standard deviation (sd.) found when registering the Quigley and Hine scores in quadrant 1 (Table 2), where plaque was abundant. The sd. was more in the normal range – but still almost doubled those of Løe and Silness’ - when registering Quigley and Hine scores in quadrant 2 where plaque was reduced considerably by brushing (Table 2). In studying the effect on gingivitis and periodontal diseases it would suffice to score the plaque deposits along the gingival margin as with the Løe and Silness plaque index [12] and the coronal spread of bacteria on the tooth surface does not seem that relevant.

In the present study only the commercial CHX products were compared, and no comparison to rinsing with a negative control, like water, saline or placebo solution was performed. The decision not to use a placebo solution was based on the fact that it would be impossible to make since the taste, smell, color and osmolality are different for the three mouthwashes. Also, the study was designed to compare 2 relatively new mouthwashes (0.12% and 0.06% CHX) on the Norwegian market against the well documented effect of the positive control - the 0.2% CHX mouthwash. Therefore, testing against a negative control as water seemed unnecessary. However, recently Preus and coworkers (Unpublished Observations; HR Preus) compared a commercially available essential oil product against sterile water in the same model, the same scoring crew and population of students as in the present study. In the present study the group rinsing with 0.06% CHX showed an average plaque score of all surfaces of 0.93 ± 0.41, which is almost half of the result of the average score, 1.7 ± 0.42, found when rinsing only with water in the former study (Unpublished Observations; HR Preus). Although the results cannot be compared directly, because they were not obtained in the same persons at the same time, it should be emphasized that in the present study the groups rinsing with CHX 0.12% and 0.06% presented with a much lower plaque score than those rinsing with water in the above mentioned, previous study (Unpublished Observations; HR Preus).

Another finding was that the commercially available 0.2% CHX mouthwash showed an average score of 0.65 ± 0.42 in the present study, being double the plaque score of what a 0.2% CHX solution produced (0.3 ± 0.2) in another comparable population and study of Preus et al. [15]. The explanation may be that in the present study the 0.2% CHX group rinsed with a commercial product, while Preus et al. [15] used a laboratory produced rinsing solution of 0.2% CHX-glukonat with 7% alcohol and 0.2% NaF in water. The reason for the self-made CHX solution proving that much better should be investigated further, but one hypothesis may be that that the 7% alcohol, which was an ingredient in the previous formulation of Corsodyl 0.2% CHX prior to 2012, may add that much to the plaque preventing effect of the product. Another may be that the added color, taste and flavor, which were not added to the laboratory produced mouthwash, may reduce the effect of the CHX in the commercial product. Regarding the known ingredient NaF, in the 0.12 and 0.06% CHX mouthwashes, it has been shown [40] that it does not reduce the effect of the CHX, at least not in dentifrices. Moreover, 0.091% NaF (in 0.12% CHX mouthwash) and 0.025% NaF (in 0.06% CHX mouthwash) is regarded as too low concentration for sufficiently preventive effect on caries [41].

There are suggestions by clinicians that lower concentrations of CHX in products may be compensated by increasing rinsing volume. In this study all the test subjects rinsed with 10 ml regardless of which product they tested. When one uses a plaque preventing rinse, it is not so much the product concentration that is interesting; it is the number of active molecules available for plaque prevention that is of the essence. Therefore, it would be wrong to compare the three products, with different concentrations, but at the same time make more of the active ingredient available by increasing the rinsing volume in the lower concentration products (0.12% and 0.06% CHX). Therefore, to perform a just comparison, they all rinsed with 10 ml of product, regardless of CHX concentration.

Among the self-reported side-effects, “loss of taste”/“taste perturbation” and “numb feeling” were the most common complaints, and the number of participants complaining about “loss of taste” was statistically significantly higher in the 0.2% CHX group than in those of 0.12% and 0.06% CHX groups. This is in concert with previous studies but in opposition to others (10).

In quadrant 2 (brushing + CHX), the plaque index remained low in all groups, suggesting that tooth brushing is sufficient to keep plaque and gingivitis scores sufficiently low to prevent gingivitis, which is in agreement with other studies [15, 41, 42]. Thus, sufficient oral hygiene for prevention of oral plaque-related diseases could easily be achieved without the help of daily antibacterial mouth rinses.

The subjects in the present study were dental hygienist, dental and medical students. One may therefore assume that they can present with a better oral hygiene than the layman. However, by comparing Q1 with mouth guard and Q2 without mouthguard in the same subjects, possible distinction from the general population was eliminated. The population in the study did not use tobacco of any kind, thus staining or masked gingival inflammation or keratinization due to tobacco did not influence on the results.

The research team was masked to the group allocation. Although both the commercial products 0.06% and 0.12% was fairly new to the market, some of the students might themselves have known the products by recognizing taste, especially those who rinsed with the commercially available 0.2% CHX mouthwash. To investigate a possible recognition effect among the participants they were asked at day 7, 14 and 21 if they had recognized the taste and knew which rinsing compound they were assigned to, and no guessing. The results of this showed that only a couple reported to recognize the compound they were rinsing with, which is not surprising since most of these young students never had been exposed to either of the commercial products tested. Thus, the study should be regarded as double blind.

## Conclusion

Among the commercial products, 0.2% CHX had significantly better plaque inhibiting effect than 0.12% CHX and 0.06% CHX, which showed no statistically significant difference between them in plaque prevention in this group of test persons. There were no differences in gingivitis between the groups after 3 weeks.

No clinically visible side effects were reported, except a very vague discoloration of teeth that was unevenly distributed among participants with no significant difference between groups.

## Additional files

Additional file 1: Assisted Questionnaire for weeks 1, 2, and 3. (DOCX 18 kb)

Additional file 2: Raw data from study (Ark1), with explanations (sheet1). (XLSX 33 kb)

## Abbreviations

AGB: Ayse Gul Bardakci
AMA: Anne Merete Aass
CHX: Chlorhexidine
GI: Gingival Index
HRP: Hans Ragnar Preus
MH: Maliha Haydari
OCK: Odd Carsten Koldsland
PI: Plaque index
Q: Quadrant
Q&H: Quigleyand Hine plaque index
REK: Regional Committee for Research Ethics
SD: Standard deviation
SPSS: Statistical Package for the Social Sciences.

## Footnotes

1 Corsodyl (0.2%), GSK, Norway

2 Flux Pro Klorhexidine (0.12%), Actavis, Norway

3 Corsodaily® (0.06%), GSK, Norway

4 Corsodyl 0.2% CHX has been alcohol free since 2012
