# Supplementary material for: Comparing the effect of 0.06% -, 0.12% and 0.2% Chlorhexidine on plaque, bleeding and side effects in an experimental gingivitis model: a parallel group, double masked randomized clinical trial
Source: BMC Oral Health. 2017 Aug 18;17:118. doi: 10.1186/s12903-017-0400-7 (PMC5562977; doi:10.1186/s12903-017-0400-7)
Supplement: Supplementary file 1 — Assisted Questionnaire for weeks 1, 2, and 3. (DOCX 18 kb) [file 12903_2017_400_MOESM1_ESM.docx]

# QUESTIONNAIRE at 1,2 and 3 weeks

1. Did you follow the protocol in all details this week?

**yes no**

If no – describe violation:

1. How did you find the taste of the product you rinsed with (multiple answers possible)

Bad Nauseating Strong Bitter Pleasant Refreshing

1. Did you experience discoloration of teeth and/or tongue?

**Teet: yes  no Tongue: yes no** Degree: little mild moderate Degree: little mild moderate

4. Did you experience other side effects when using your allocated product?

**Yes No**

If yes: Taste perturbation Loss of taste Numbness Dry feeling

Sensitivity loss in tongue or mucous membranes Unpleasant feeling

1. Did you recognize which product you were allocated?

Yes No

If yes – which?

**Corsodaily FluxProKlorheksidin Corsodyl**
